# Brain Natriuretic Peptide and Its Biochemical, Analytical, and Clinical Issues in Heart Failure: A Narrative Review

**DOI:** 10.3389/fphys.2018.00692

**Published:** 2018-06-05

**Authors:** Shihui Fu, Ping Ping, Qiwei Zhu, Ping Ye, Leiming Luo

**Affiliations:** ^1^Department of Geriatric Cardiology, Chinese People’s Liberation Army General Hospital, Beijing, China; ^2^Department of Cardiology and Hainan Branch, Chinese People’s Liberation Army General Hospital, Beijing, China; ^3^Department of Pharmaceutical Care, Chinese People’s Liberation Army General Hospital, Beijing, China

**Keywords:** a disintegrin and metalloprotease, B-type natriuretic peptide, chronic kidney disease, corin, furin, heart failure, LCZ696, proprotein convertase subtilisin/kexin-6

## Abstract

Heart failure (HF) is a primary cause of morbidity and mortality worldwide. As the most widely studied and commonly applied natriuretic peptide (NP), B-type natriuretic peptide (BNP) has the effects of diuresis, natriuresis, vasodilation, anti-hypertrophy, and anti-fibrosis and it inhibits the renin-angiotensin-aldosterone and sympathetic nervous systems to maintain cardiorenal homeostasis and counteract the effects of HF. Both BNP and N-terminal pro B-type natriuretic peptide (NT-proBNP) are applied as diagnostic, managing, and prognostic tools for HF. However, due to the complexity of BNP system, the diversity of BNP forms and the heterogeneity of HF status, there are biochemical, analytical, and clinical issues on BNP not fully understood. Current immunoassays cross-react to varying degrees with pro B-type natriuretic peptide (proBNP), NT-proBNP and various BNP forms and cannot effectively differentiate between these forms. Moreover, current immunoassays have different results and may not accurately reflect cardiac function. It is essential to design assays that can recognize specific forms of BNP, NT-proBNP, and proBNP to obtain more clinical information. Not only the processing of proBNP (corin/furin) and BNP (neprilysin), but also the effects of glycosylation on proBNP processing and BNP assays, should be targeted in future studies to enhance their diagnostic, therapeutic, and prognostic values.

## Introduction

Heart failure is a primary cause of morbidity and mortality worldwide. As the most widely studied and commonly applied NP, BNP has the effects of diuresis, natriuresis, vasodilation, anti-hypertrophy and anti-fibrosis and it inhibits the renin-angiotensin-aldosterone and sympathetic nervous systems to maintain cardiorenal homeostasis and counteract the effects of HF (Semenov and Katrukha, 2016). Both BNP and NT-proBNP are established as HF biomarkers and recommended by international guidelines (Thygesen et al., 2012; Fu et al., 2018). Although affected by age, gender, obesity, renal function, and other factors, plasma BNP levels are closely related to HF severity and applied as diagnostic, managing and prognostic tools for HF (Vasile and Jaffe, 2017). However, due to the complexity of BNP system, the diversity of BNP forms and the heterogeneity of HF status, there are biochemical, analytical and clinical issues on BNP not fully understood (Iwaz and Maisel, 2016).

## Biochemical Issues

**FIGURE 1:**
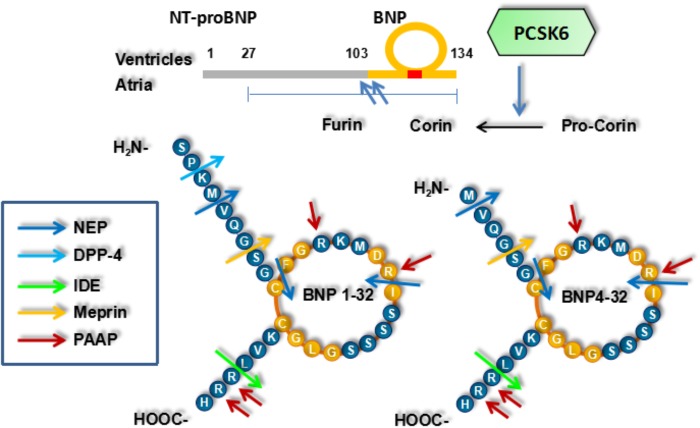
Biochemical issues of brain natriuretic peptide.

The 134-aa preproBNP is synthesized in the cardiomyocytes, and 108-aa proBNP is then produced by removing a 26-aa signal peptide (**Figure 1**). Enzyme-mediated processing of proBNP produces BNP (32-aa) with mature function and NT-proBNP (76-aa), which are released into the blood when the ventricular wall is stretched because of increased pressure or volume overload (Palazzuoli et al., 2010; Semenov et al., 2010). proBNP is degraded into NT-proBNP and BNP in a 1:1 ratio. NT-proBNP has no known bioactivity and is present at higher levels than BNP, perhaps due to slower clearance from blood (Bayes-Genis et al., 2004).

Corin and furin are the possible proBNP activating enzymes produced in the cardiomyocytes and both the soluble circulating and membrane-bound forms may be involved in proBNP processing (Clerico et al., 2011). Corin and furin are present at the cell surface with the ability to process proBNP and have soluble forms in blood raising the possibility of proBNP processing in blood (Jiang et al., 2011). Corin and furin may produce different BNP forms: BNP4-32 and BNP1-32, respectively (Semenov et al., 2011). However, other enzymes cannot be excluded in humans (Koo et al., 2006; Gladysheva et al., 2008). Soluble corin is significantly decreased in HF, reminding us that it may have an attenuated activation and be applied as a biomarker in HF (Miyazaki et al., 2016). Moreover, corin and furin activation may correlate with BNP bioactivity, whereas their deficiency may correlate with hypertension and HF (Chan et al., 2005). Corin and furin overexpression may be beneficial in experimental HF models. Unprocessed proBNP has higher levels in patients with HF, suggesting that their activities are rate-limiting factors in HF (Gladysheva et al., 2013). The various BNP forms have different cGMP activating properties and proBNP and NT-proBNP have reduced cGMP activities (Heublein et al., 2007; Dong et al., 2012). However, there have been almost no studies with the specific purpose of assessing proBNP processing in blood (Clerico et al., 2015a). proBNP processing is significantly disturbed in HF and may be a novel target for drugs (Del Ry et al., 2013; Clerico et al., 2015a).

A disintegrin and metalloprotease 10 mediates corin shedding and decreases corin bioactivity at the cell surface (Jiang et al., 2011). In humans, corin is expressed not only in the heart, but also in the kidney (proximal convoluted tubules and medullary collecting ducts) (Ichiki et al., 2011). In proximal tubular epithelial cells, corin is expressed in the apical membrane, whereas neprilysin is expressed in the brush border (Dong et al., 2016). Reduced renal corin expression and urine soluble corin in CKD may prevent local function of NPs (Fang et al., 2013). Reduced renal NP autocrine may contribute to NP resistance and further disturb cardiorenal homeostasis, if it is not compensated by increased cardiac NP endocrine (Dong et al., 2016). ADAM 10-mediated shedding may reduce corin levels in the kidney. Future studies are needed to analyze renal corin expression and ADAM 10-mediated shedding in patients with HF and/or CKD.

Proteolytic cleavage (Arg801-Ile802) may activate corin, with PCSK6 as an activating enzyme (Chen et al., 2015; Volpe and Rubattu, 2016). Among nine members of proprotein convertase subtilisin/kexin (PCSK) family, PCSK6 overexpression can enhance corin activation. Meanwhile, selective PCSK6 gene silencing by small interfering RNAs can abolish corin activation. PCSK6 gene expression can be detected in cells expressing corin, and corin variants without the cleavage site for PCSK6 are resistant to PCSK6 activation (Wang et al., 2008). However, previous studies have demonstrated that not only proBNP levels, but also cardiac function and hypertrophy, cannot be changed by affecting either corin or PCSK6, in spite of reduced proANP levels and effective control of hypertension (Chen et al., 2015). PCSK family includes a series of serine endoproteases with many substrates. Other substrates of PCSK6 are cytokines of TGF-β family, nodal growth differentiation factor pro-protein and aggrecanases (Turpeinen et al., 2013). Atrial natriuretic peptide (ANP) has an anti-hypertrophic effect on the heart through TGF-β signaling (Calvieri et al., 2012). PCSK6 may inhibit the cytokines of TGF-β family, and counteract the anti-hypertrophic effect of ANP. Another member of PCSK family, PCSK9, mediates degradation of low-density lipoprotein cholesterol and has been recommended as a target for a novel lipid-lowering drug (Navarese et al., 2015). As a corin activating enzyme, PCSK6 may be a novel target for drugs in HF by mediating proBNP degradation and increasing endogenous BNP (Volpe et al., 2016).

B-type natriuretic peptide is degraded by neprilysin, dipeptidyl peptidase-4 (DPP-4) and insulin-degrading enzyme (IDE) (Ralat et al., 2011). Peptidyl arginine aldehyde protease has been shown to degrade BNP at the sites with arginine, because its inhibitors reduce BNP degradation (Belenky et al., 2004). Meprin has been shown to degrade BNP in animals but not in humans. Whether other enzymes, including meprin, degrade BNP remains undetermined in humans (Dickey and Potter, 2010). BNP is degraded by neprilysin at several sites, but not at these sites simultaneously. proBNP differs from BNP with a 76-aa N-terminal extension and may not be a substrate of neprilysin, suggesting potential effects of NP length and N-terminal extension on neprilysin degradation (Pankow et al., 2009). Similarly, urodilatin is a N-terminal extended form of ANP and less rapidly degraded than ANP. D-type NP is not degraded and has the longest extension among NPs (Pankow et al., 2009). Neither glycosylated nor non-glycosylated forms of proBNP are sensitive to neprilysin degradation, suggesting no effect of glycosylation on proBNP resistance to neprilysin. Moreover, BNP may be a poorer substrate of neprilysin than ANP (Dickey and Potter, 2011). However, neprilysin has inconsistent effects on BNP degradation, perhaps because of different experimental conditions and race specificities.

Both proBNP and NT-proBNP are glycosylated in blood and the potential glycosylation sites are Thr36, Ser37, Ser44, Thr48, Ser53, Thr58, and Thr71 within the N-terminal region (aa residue 1–76), but not within the BNP (aa residue 77–108) (Seferian et al., 2008). All these glycosylation sites are complete except Thr36 and Thr58 (Schellenberger et al., 2006). NT-proBNP is glycosylated in the central region (aa residue 28–56), but not in the C-terminal region (aa residue 61–76) (Seferian et al., 2008). proBNP is glycosylated not only in the central region, but also in the region near the cleavage site (aa residue 63–76) (Semenov et al., 2009). Percentages of glycosylated proBNP and NT-proBNP are dependent on the individual. Patients with chronic HF, but not those with acute HF, have the highest percentage of glycosylated proBNP (Vodovar et al., 2014). Meanwhile, furin bioactivity, but not its levels, is greater in patients with acute HF than in those with chronic HF (Vodovar et al., 2014). proBNP processing may have different mechanisms: patients with acute HF have increased BNP production due to more acute fluid overload and patients with chronic HF have limited proBNP degradation due to less acute fluid overload. Glycosylation, especially at the Thr71 near the cleavage site, may inhibit corin- and furin-mediated degradation of proBNP in HF (Schellenberger et al., 2006). This effect remains undetermined but may correlate with whether proBNP is processed in blood (Peng et al., 2011; Halfinger et al., 2017).

## Analytical Issues

Current NT-proBNP immunoassays have the same antibodies and calibrators from Roche^TM^ with small systematic differences (Clerico et al., 2012). However, current BNP immunoassays (**Table 1**) have different antibodies and calibrators with large systematic differences (Clerico et al., 2005). The most common BNP immunoassays are sandwich immunoassays with two monoclonal or polyclonal antibodies binding to two separate epitopes: one binds to the ring structure to recognize the active form and the other binds to the N-terminal or C-terminal region (Franzini et al., 2013). The one binding to the C-terminal region [Shionogi^TM^ IRMA and Siemens ADVIA for the Centaur platform] with same monoclonal antibodies (the epitope 27–32 and 14–21) may not recognize cleaved forms of BNP like BNP1-27, while the one binding to the N-terminal region [Alere^TM^ and Beckman Coulter^TM^ Triage BNP assays with the same monoclonal (the epitope 5–13) and polyclonal (the possible epitope 1–10) antibodies] may not recognize cleaved forms of BNP like BNP3-32 (Belenky et al., 2004). The single-epitope sandwich (SES)-BNP^TM^ immunofluorescent assay needs only one epitope by two different monoclonal antibodies, including the first monoclonal antibody (24C5) binding to the epitope 11–17, which is the most stable within the ring structure, and the second monoclonal antibody (Ab-BNP2) binding to the immune complex (the epitope 11–17 and 24C5) (Tamm et al., 2008). As a highly sensitive assay, it stabilizes the immune complex and increases epitope affinity but recognizes not only BNP forms, but also glycosylated and non-glycosylated forms of proBNP.

**Table 1:** Antibodies and standard materials used in commercial B-type natriuretic peptide (BNP) immunoassays.

Methods	Capture antibody	Detection antibody	Standard material
Shionogi^TM^ immunoradiometric assay (IRMA)	COOH terminus (BC-203), murine monoclonal AB, aa 27–32	Ring structure (KY-hBNPII) (Shionogi), murine monoclonal AB	Synthetic BNP
Siemens^TM^ ADVIA method for Centaur platform	COOH terminus (BC-203), murine monoclonal AB, aa 27–32	Ring structure (KY-hBNPII) (Shionogi), murine monoclonal AB	Synthetic BNP
Alere^TM^ Triage	NH_2_ terminus and part of the ring structure (Scios), murine monoclonal AB, aa 5–13	BNP (Biosite), murine omniclonal AB, epitope not characterized (probably N-terminus 1–10)	Recombinant BNP
Beckman Coulter^TM^ Triage	BNP (Biosite), murine omniclonal AB, epitope not characterized (probably N-terminus 1–10)	NH_2_ terminus and part of the ring structure (Scios), murine monoclonal AB, aa 5–13	Recombinant BNP
Abbott^TM^ i-STAT	NH_2_ terminus and part of the ring structure (Scios), murine monoclonal AB, aa 5–13	COOH terminus, murine monoclonal AB, aa 26–32	Synthetic BNP

*aa, amino acid*.

Current BNP immunoassays substantially reflect total levels of proBNP and BNP forms (Liang et al., 2007). Although proBNP processing occurs before or during secretion, unprocessed proBNP is present in blood at even higher levels than BNP and represents a significant part of BNP immunoreactivity in healthy individuals and HF patients (Costello-Boerrigter et al., 2013). Corin and furin cannot process all the proBNP when proBNP production is obviously increased in patients with HF, or proBNP glycosylation occurs before secretion, especially at the Thr71 (Clerico et al., 2015a). Because proBNP shares a 32-aa structure with BNP, proBNP can mediate physiological functions like BNP. However, as the predominant form of BNP immunoreactivity in HF, unprocessed proBNP has an obviously decreased physiological function compared with BNP (Liang et al., 2007). Meanwhile, due to the cleavage of amino-terminal dipeptide from BNP1-32 by DPP-4 and neprilysin, BNP3-32 and 5–32 are present in blood. BNP3-32 rather than BNP1-32 may be the predominant form of BNP (Suzuki et al., 2017). Compared with BNP1-32, other BNP forms, such as BNP3-32, BNP4-32, BNP5-32, BNP5-31, BNP1-27, BNP 1-26, and BNP 1-25, have an obviously decreased physiological function and increased degradation rate by neprilysin (Brandt et al., 2006). However, due to race-specific proteases, BNP forms may have no effect on the resistance of human BNP to neprilysin (Brandt et al., 2006). Current BNP immunoassays overestimate BNP1-32 levels, because they also recognize less active BNP forms (Lewis et al., 2017).

Antibody detection of NT-proBNP and proBNP may also be affected by glycosylation (Peng et al., 2011). Glycosylation suppresses binding of antibodies and makes them lose immunoreactivity (Luckenbill et al., 2008). Current NT-proBNP immunoassays cross-react with non-glycosylated proBNP, and do not detect glycosylated NT-proBNP and proBNP. NT-proBNP immunoassays may be improved by antibodies detecting glycosylated or non-glycosylated NT-proBNP and antibodies detecting NT-proBNP not affected by glycosylation (Rosjo et al., 2015). In current proBNP immunoassays, glycosylated proBNP cross-reacts more than non-glycosylated proBNP with BNP and NT-proBNP (Emdin et al., 2011). There are inter-individual differences in NT-proBNP and proBNP glycosylation in patients with and without HF (Saenger et al., 2017). The N- and C-terminal regions of NT-proBNP and BNP are degraded in blood, which occurs between Pro2-Leu3, Leu3-Gly4, Pro6-Gly7, and Pro75-Arg76 of NT-proBNP (Foo et al., 2013). Thus, detecting N- and C-terminal cleaved forms of BNP and NT-proBNP is another challenge and it is difficult to develop an antibody not affected by glycosylation or terminal cleavage.

A highly sensitive immunoassay for proBNP is not affected by proBNP glycosylation, because it has a capture monoclonal antibody binding to the epitope 26–32 of BNP and a detection monoclonal antibody binding to the epitope 13–20 of proBNP, neither of which are glycosylation sites (Seferian et al., 2007). Another immunoassay for proBNP has no significant cross-reaction with both NT-proBNP and BNP, because it has a polyclonal antibody binding to BNP and a monoclonal antibody binding to the cleavage site of proBNP, an epitope only belonging to proBNP (Macheret et al., 2011). Meanwhile, a radioimmunoassay for proBNP binds to the N-terminal of proBNP (the epitope 1–10) and recognizes both proBNP and NT-proBNP (Goetze et al., 2002). Automated immunoassays specific for both proBNP and BNP1-32 may be useful to determine both production and bioactivity of BNP forms. Moreover, proBNP and BNP immunoassays have been combined to better predict poor prognosis in patients with HF (Dries et al., 2010). Two immunoassays can be simultaneously applied to the same sample. However, BNP1-32 immunoassays with chromatography and mass spectrometry are unsuitable for routine application and there is no commercially available immunoassay that can recognize only active BNP1-32 (Miller et al., 2011).

Neprilysin inhibition may have varied effects on plasma BNP levels as a result of different immunoassays. The N-terminal Met4-Phe5 is the initial cleavage site and no BNP immunoassay has any antibody binding to it. Another cleavage site is located within the ring structure (Arg17-Ile18), which is cleaved before other sites, such as Lys14-Met15, Gly23-Leu24, and Val28-Leu29, and BNP immunoassays (Shionogi^TM^ and Siemens^TM^) with antibodies binding to the epitopes 14–21 may be sensitive to neprilysin degradation (Clerico et al., 2015b). With only one epitope and without space between epitopes, SES-BNP^TM^ assay is not sensitive to neprilysin degradation.

Blood samples for BNP assays should be drawn only in plastic tubes because BNP is unstable in glass tubes due to kallikrein activation (Apple et al., 2005). EDTA plasma is the only recommended specimen for BNP assays and serum is the recommended specimen for NT-proBNP assays. There are significant differences between serum and plasma levels of NPs with various detection platforms. Anticoagulant type is also significant. BNP is stable during storage at room temperature for 24 h or at 30°C for 12 h. Protease inhibitor (aprotinin) can be added to increase BNP storage time. NT-proBNP is stable during storage in serum, heparinized plasma or EDTA plasma at room temperature or at 4°C for 72 h or at -80°C for up to 1 year (Dong et al., 2012). It is essential to validate the effect of freeze-thaw cycles on the stability of BNP and NT-proBNP assays (Apple et al., 2005).

## Clinical Issues

Point of care testing is performed near the patients outside of the central laboratory with a rapid turnaround (Iwaz and Maisel, 2016). POCT for BNP and NT-proBNP can effectively facilitate not only home-monitoring and community-service outside of hospitals, but also emergency testing and BNP-guided therapy in hospitals (Christenson et al., 2014). However, due to poor performance (particularly sensitivity and precision) compared with laboratory assays, POCT for BNP and NT-proBNP has not been finally approved by authoritative organizations and is not widely available for clinical application (Jungbauer et al., 2012). POCT for BNP with untreated fingertip capillary whole blood (Alere^TM^ Heart Check) closely correlates with POCTs for BNP with venipuncture EDTA plasma (Alere^TM^ Triage) or EDTA whole blood (Abbott^TM^ i-STAT) (Maisel et al., 2013; Prontera et al., 2015). Abbott^TM^ i-STAT produces a result within 10 min, whereas Alere^TM^ Triage and Alere^TM^ Heart Check produces their results within 15 min. It remains undetermined whether POCTs have good correlation with and similar precision to laboratory assays (Shah et al., 2010). Abbott^TM^ i-STAT and Alere^TM^ Triage reduce HF diagnosis compared with laboratory assays (Kosowsky et al., 2006). Both POCT and laboratory assays have systematic differences caused by cross-reaction with glycosylated or non-glycosylated forms (Clerico et al., 2015b).

In the emergency room, rapid assay for BNP and NT-proBNP can discriminate the origin of acute dyspnea (acute HF versus bronchial asthma) (Fu et al., 2018). A plasma NT-proBNP level of 300 pg/ml is appropriate for ruling out acute HF. Age-dependent cutoff levels of plasma NT-proBNP are appropriate for ruling in acute HF: 450 pg/ml in patients <50 years of age, 900 pg/ml in patients ≥50 years of age, and 1800 pg/ml in patients >75 years of age (McMurray et al., 2012). Plasma BNP levels of 100 and 400 pg/ml are appropriate for ruling out and ruling in acute HF, respectively (Dickstein et al., 2008).

The recombinant form of BNP (nesiritide) has been applied as a conventional drug in HF. It is currently considered to improve clinical symptoms and cardiac function and has no effect on patient prognosis (Fu et al., 2012). An angiotensin receptor inhibitor and neprilysin inhibitor (ARNI, LCZ696) has been approved as a novel drug in HF by US Food and Drug Administration (McMurray et al., 2013). Because LCZ696 affects plasma BNP levels through inhibiting BNP degradation by neprilysin, it makes plasma BNP levels not accurately reflect cardiac function and produces a challenge for using BNP as a biomarker, making HF diagnostically ambiguous and therapeutically misleading (Packer et al., 2015). It could be argued that NT-proBNP is insensitive to neprilysin degradation and therefore can be applied as a biomarker in patients with LCZ696. However, this assumption is based on a simplified model of a complex biological phenomenon, and more studies are essential to analyze this biological phenomenon. Due to the complexity of BNP system (proBNP and BNP forms), neprilysin inhibition does not have a straightforward effect on plasma BNP levels (Pemberton et al., 2012). It remains undetermined how LCZ696 affects plasma BNP and NT-proBNP levels. Prospective comparison of ARNI with ACEI to Determine Impact on Global Mortality and morbidity in Heart Failure (PARADIGM-HF) trial has shown that plasma BNP levels increase while plasma NT-proBNP levels decrease in patients with LCZ696. Angiotensin receptor inhibitor and neprilysin inhibitor (ARNI, LCZ696) may inhibit both BNP degradation and proBNP processing. Increased BNP levels may inhibit proBNP production and thus decreased NT-proBNP levels may not be caused by improved cardiac function. Moreover, beneficial effects of LCZ696 in HF may be accomplished by increasing plasma ANP and C-type natriuretic peptide (CNP) levels but not plasma BNP levels. However, considering the presence of BNP in blood and its sensitivity to neprilysin, a relatively modest increase in plasma BNP levels may also account for improved prognosis with LCZ696 in patients with HF (McMurray et al., 2014). Several studies have realized improved mortality and admission with BNP-guided therapy (Valle et al., 2011). However, previous studies have yielded inconsistent results on NT-proBNP-guided therapy (Sanders-van Wijk et al., 2014). Current randomized clinical trials on BNP-guided therapy may further evaluate BNP-guided therapy in patients with HF (Januzzi and Troughton, 2013).

## Conclusion

B-type natriuretic peptide metabolism and its forms are complex and make assays particularly challenging, but critical to providing future insight into BNP application and HF heterogeneity. Current immunoassays cross-react to varying degrees with proBNP, NT-proBNP, and various BNP forms and cannot effectively differentiate between these forms. Moreover, current immunoassays have different results and may not accurately reflect cardiac function. It is essential to design assays that can recognize specific forms of BNP, NT-proBNP, and proBNP to obtain more clinical information. Considering the complexity of BNP system and the heterogeneity of HF status, not only the processing of proBNP (corin/furin) and BNP (neprilysin), but also the effects of glycosylation on proBNP processing and BNP assays, should be targeted in future studies to enhance their diagnostic, therapeutic and prognostic values.

## Author Contributions

SF, PP, QZ, PY, and LL contributed to the study design, performed data collection and analyses, and drafted this paper.

## Conflict of Interest Statement

The authors declare that the research was conducted in the absence of any commercial or financial relationships that could be construed as a potential conflict of interest.

## Abbreviations

aa: amino acid
ADAM: a disintegrin and metalloprotease
ARNI: angiotensin receptor inhibitor and neprilysin inhibitor
BNP: B-type natriuretic peptide
CKD: chronic kidney disease
EDTA: ethylenediaminetetraacetic acid
HF: heart failure
IRMA: immunoradiometric assay
NP: natriuretic peptide
NT-proBNP: N-terminal pro B-type natriuretic peptide
PCSK6: proprotein convertase subtilisin/kexin-6
POCT: point of care testing
proANP: pro-atrial natriuretic peptide
proBNP: pro B-type natriuretic peptide
TGF-β: transforming growth factor-β.
